# DNA Aptamers Selectively Target *Leishmania infantum* H2A Protein

**DOI:** 10.1371/journal.pone.0078886

**Published:** 2013-10-21

**Authors:** M. Elena Martín, Marta García-Hernández, Eva M. García-Recio, Gerónimo F. Gómez-Chacón, Marta Sánchez-López, Víctor M. González

**Affiliations:** 1 Departamento de Bioquímica-Investigación, Instituto Ramón y Cajal de Investigación Sanitaria, Hospital Ramón y Cajal, Madrid, Spain; 2 Aptus Biotech SL, Madrid, Spain; Louisiana State University, United States of America

## Abstract

Parasites of the genus *Leishmania* produce leishmaniasis which affects millions people around the world. Understanding the molecular characteristics of the parasite can increase the knowledge about the mechanisms underlying disease development and progression. Thus, the study of the molecular features of histones has been considered of particular interest because *Leishmania* does not condense the chromatin during mitosis and, consequently, a different role for these proteins in the biology of the parasite can be expected. Furthermore, the sequence divergences in the amino and in the carboxy-terminal domains of the kinetoplastid core histones convert them in potential diagnostic and/or therapeutics targets. Aptamers are oligonucleotide ligands that are selected *in vitro* by their affinity and specificity for the target as a consequence of the particular tertiary structure that they are able to acquire depending on their sequence. Development of high-affinity molecules with the ability to recognize specifically *Leishmania* histones is essential for the progress of this kind of study. Two aptamers which specifically recognize *Leishmania infantum* H2A histone were cloned from a previously obtained ssDNA enriched population. These aptamers were sequenced and subjected to an *in silico* analysis. ELONA, slot blot and Western blot were performed to establish aptamer affinity and specificity for LiH2A histone and ELONA assays using peptides corresponding to overlapped sequences of LiH2A were made mapping the aptamers:LiH2A interaction. As “proofs of concept”, aptamers were used to determine the number of parasites in an ELONA platform and to purify LiH2A from complex mixtures. The aptamers showed different secondary structures among them; however, both of them were able to recognize the same peptides located in a side of the protein. In addition, we demonstrate that these aptamers are useful for LiH2A identification and also may be of potential application as diagnostic system and as a laboratory tool with purification purpose.

## Introduction

Leishmaniasis consists of a pool of parasitic illnesses provoked by several species of the kinetoplastid parasite *Leishmania*. The disease is endemic in 88 countries on 4 continents affecting 12 million people around the world with an annual death rate of approximately 80,000 people [1,2]. Over 20 *Leishmania* species and subspecies infect humans, each causing a different spectrum of symptoms. These range from simple, self-healing skin ulcers (e.g. due to infection with *L. major*), to severe, life-threatening disease (e.g. visceral leishmaniasis caused by *L. donovani*). Humans are infected via the bite of sandflies (subfamily *phlebotominae*) that breed in forest areas, caves, or the burrows of small rodents. Wild and domesticated animals and humans themselves can act as a reservoir of infection. Most forms of leishmaniasis are originally infections of small mammals (‘reservoir hosts’), which play a major role in the epidemiology of the disease. Sandflies become infected by ingesting blood from infected reservoir hosts or from infected people.

*Leishmania* parasites possess a digenetic life cycle with two discrete morphological phases: the promastigote, which develops extracelullarly within the gut of the insect vector, and the amastigote that is specialized to survive within the macrophage phagolysosome of vertebrate host [3]. These parasites, like other related kinetoplastid protozoa, are placed in the most primitive branch of the eukaryote evolution and possess very peculiar features of gene expression and organization. Among them, the organization of the nuclear genome differs from that of higher eukaryotes. For example, the nuclear envelope persists during cell division, chromosomes are not visualized at any phase of the cell cycle. In addition, chromatin is organized in nucleosomes but higher-order structures of 30 nm fibers are not observed. Interestingly, although histones are extremely conserved proteins, reflecting their apparent universality of function, the sequence divergences in the amino and in the carboxy-terminal domains of the histones convert them into potential diagnostic and/or therapeutics targets. This fact makes the search for molecules that specifically recognize *Leishmania* histones of great interest.

Aptamers are single-stranded (ss) oligonucleotides which are selected from combinatorial libraries by systemic evolution of ligands using exponential enrichment (SELEX) technology and are capable of selectively binding target molecules with high affinity [4,5]. Aptamers acquire unique three-dimensional structures due to the ability of short sequences to fold in the presence of a ligand. These highly structured aptamers are capable to bind to the target with high affinity and specificity. Structural studies with aptamer-target complexes have demonstrated insights into molecular diversity associated with nucleic acid architecture and molecular recognition [6]. Thus, it has been shown that aptamers form stable and specific complexes with a range of different targets including small molecules such as amino acids to highly complex proteins and whole viruses [7-14].

After a SELEX process, a population enriched with sequences that display high affinity (in the nanomolar or subnanomolar range) and specificity for the target is obtained. This final aptamer population may act like polyclonal antibodies whereas individual cloned aptamers may mimic monoclonal antibodies. Although aptamers are similar to antibodies in specificity and affinity, they have many potential advantages due to their nucleotidic nature [15]. Accordingly, aptamers are reproduced by chemical synthesis, their smaller size allows more efficient entry into biological compartments, and they are non-immunogenic. In addition, aptamers are easily labeled with fluorescent or other reporters during their synthesis [16], they can be chemically modified to become extremely stable [17,18], or can be further truncated to eliminate oligonucleotide sequences which are not important for the interaction with the target or for the correct tridimensional aptamer structure [19,20]. Consequently, they are considered potential diagnostic and therapeutic agents and also thought to be rival antibodies in immunoassay-like analyses.

In addition to the potential therapeutic and/or diagnostic use of the aptamers, these molecules can be used for ligand-mediated purification and identification of their targets on cell membranes [21]. Recently, several authors reviewed an approach for the development of aptamers targeting surface proteins of *Trypanosoma* and *Plasmodium* and their potential applications in diagnostic systems [22,23].

During the last years, we have dedicated a lot of effort to the selection of aptamers that specifically recognize *Leishmania* proteins in order to obtain investigation tools and potentially develop a detection system for leishmaniasis. At this respect aptamer populations targeting *L. infantum* KMP-11[24,25], LiH2A [26] and LiH3 [27] have been selected and characterized in our laboratory. Thus, we isolated a pool of aptamers of ssDNA sequences, named SELH2A, which specifically binds to *L. infantum* histone H2A [26]. When tested in enzyme-linked oligonucleotide assay (ELONA), slot blot and Western blot, the aptamer pool exhibited specificity in its ability to bind only to H2A antigen but not to other proteins from *L. infantum* including other histones. In this paper we detail the isolation and characterization of two aptamers against LiH2A that are able to recognize specifically the protein into complex mix and permits purify LiH2A from fractions enriched in this protein.

## Materials and Methods

### Reagents

Chemical products were obtained from Sigma-Aldrich (Spain) except those indicated in the text. RND40 starting population, individuals DNA aptamers, and their derivatives were purchased from IBA GmbH (Göttingen, Germany).

### Cell culture and extract preparation

*Leishmania infantum* (MCAN/ES/96/BCN150) promastigotes were grown at 26°C in RPMI 1640 medium (PAA Laboratories), supplemented with 10% (v/v) foetal calf serum (PAA Laboratories), 10 U/ml penicillin and 100 μg/ml streptomycin (Gibco). Total protein extracts were obtained from *L. infantum* promastigotes that were pelleted and lysed in 125 mM Tris–HCl pH 6.8, 4% SDS, 15 mM EDTA, 20% glycerol, 10% β-mercaptoethanol, and 0.008% bromophenol blue. Cultures containing 1.4x10^7^ promastigotes/ml were pelleted were lysed in ice-cold buffer A (20 mM Tris–HCl pH 7.6, 1 mM dithiothreitol (DTT), 1 mM EDTA, 1 mM phenylmethanesulfonyl fluoride (PMSF), 1 mM benzamidine, 2 mM sodium molybdate, 2 mM sodium β-glycerophosphate, 0.2 mM sodium orthovanadate, 120 mM KCl, 1 μg/ml leupeptin and pepstatin A, and 10 μg/ml antipain) containing 1% Triton X-100 and then centrifuged at 17000 x g for 20 min in order to obtain a cytoplasmic fraction. The pellet was resuspended in RIPA buffer (20 mM Tris-HCl pH 7.6, 150 mM NaCl, 1 mM EDTA, 1 mM EGTA, 1% NP-40, 1% sodium deoxycholate, 2.5 mM sodium pyrophosphate, 1 mM β-glycerophosphate, 1 mM sodium orthovanadate, 1 μg/ml leupeptin) and centrifugated at 17000 x g for 20 min, to obtain a fraction enriched in nuclear proteins. Protein determination was performed by the method of Bradford [28]. The volume of both supernatants was accurate measured to calculate that corresponding to 10^6^ promastigotes.

### Expression and purification of recombinant *Leishmania infantum* H2A

Recombinant *L. infantum* H2A (rLiH2A) protein was expressed and purified by affinity chromatography on Ni-NTA resin columns as described [26]. To obtain cells expressing LiH2A in native conditions, H2A expression were induced with 1 mM IPTG (Sigma) during 2 h and cells were harvested, resuspended in lysis buffer (5 mM sodium phosphate, 150 mM NaCl, 1 mM EDTA, 0.5% Triton X-100, pH 7.5), sonicated on ice, and supernatant was obtained by centrifugation at 17000 x g for 20 min.

### Aptamer cloning and sequencing and secondary ssDNA structure prediction

An aptamer population against rLiH2A was previously selected [26]. Briefly, ssDNA oligonucleotides, designated as RND40, contained a central randomized region of 40 nucleotides flanked by two conserved 18-nucleotides regions in each end (5´-GCGGATGAAGACTGGTCT-40N-GTTGCTCGTATTTAGGGC-3´). For the initial SELEX round, 2 nmol of ssDNA, denatured at 90°C for 10 min and then cooled on ice for 10 min, were mixed with 2 µg of rLiH2A in 200 µl of selection buffer (20 mM Tris-HCl, pH 7.4, 1 mM MgCl_2_, 150 mM NaCl, 5 mM KCl, 0.2% BSA) and incubated at 37°C for 30 min. The bound aptamer–H2A complexes were purified using Ni-NTA superflow (Qiagen) and the ssDNA bound to the protein were amplified by PCR using the primers named F3 (5´-GCGGATGAAGACTGGTGT-3´) and R3 (5´- GTTGCTCGTATTTAGGGC-3´) [26].

Selected aptamer population was amplified by using 1U Taq DNA polymerase (Biotools) in 50 µL of reaction also containing 1xPCR buffer (75 mM Tris HCl pH 9.0, 50 mM KCl, 20 mM (NH_4_)_2_SO_4_, 2 mM MgCl_2_, 125 mM dNTPs, 1 µM F3 primer and 1 µM R3 primer. The dsDNA product with ‘A’-overhangs was cloned into pGEM-T Easy-cloning vector (Promega) following manufacturer’s instructions. Individual clones were sequenced using T7 (5´-TAATACGACTCACTATAGGG-3´) and Sp6 (5´- ATTTAGGTGACACTATAGAA-3´) primers (IBA GmbH).

Selected ssDNA molecules were subjected to secondary structure prediction using the mFold software (http://mfold.rna.albany.edu/?q=mfold/DNA-Folding-Form) [29] at 26°C in 150 mM [Na^I^] and 1 mM [Mg^II^] and QGRS Mapper, a web-based server for predicting G-quadruplexes in nucleotide sequence (http://bioinformatics.ramapo.edu/QGRS/analyze.php).

### Kinetics and binding capacity and specificity studies by Enzyme-Linked OligoNucleotide Assay (ELONA)

ELONA was used to analyze the affinity and specificity of the aptamers for the target as in Ramos et al., 2007 [26]. First, in order to study kinetics of binding of aptamers to LiH2A, *L. infantum* H2A (rLiH2A) protein was diluted at 5 µg/mL (330 nM) in selection buffer (20 mM Tris-HCl, pH 7.4, 1 mM MgCl_2_, 150 mM NaCl, 5 mM KCl) with 0.2% BSA and 200 μL of the solution (1 µg/well, 66 pmol/well) were incubated in a 96-well microtiter plate (NUNC) overnight at 4°C. Afterwards, digoxigenin-labeled aptamers (IBA GmbH) were diluted in selection buffer at 500 ng/mL (20 nM), denatured for 10 min at 95°C and cooled for 10 min on ice. Then, 200 µL of the solution were added to each well, the plate incubated at 26°C for several incubation times after which individual wells were washed four times with selection buffer to remove unbound ssDNA. Next, 200 µL of a 1/1000 dilution of anti-digoxigenin antibody conjugated with horse-radish peroxidase (POD) (Roche) were added to the individual wells. Following 30 min incubation at room temperature on a shaking platform, the plates were washed four times and developed using ABTS solution (Boehringer Mannheim) according to the manufacturer’s instruction. OD values at 405 nm were determined using a microplate reader from TECAN.

In order to determine the lowest limit of protein detected by aptamers, several amounts of rLiH2A (0-800 ng/well; 0-52.8 pmol/well) were incubated with 200 µL of digoxigenin-labeled aptamers diluted at 125 ng/mL (5 nM), previously structured as indicated above. Afterwards, the plate was incubated at 26°C for 30 min and then washed four times with selection buffer. Next, anti-digoxigenin-POD antibody was added and developed using ABTS solution as above.

To determinate the constant affinity of the aptamers for rLiH2A, 200 ng/well (13.2 pmol/well) of rLiH2A protein were plated in coating solution (KPL) and incubated in a 96-well microtiter plate overnight at 4°C. Then, the wells were blocked 1 h with BSA 5% in PBS and washed four times in selection buffer. Afterwards, biotin-labeled aptamers (IBA GmbH) were diluted in selection buffer at concentration between 0-50 nM, denatured for 10 min at 95°C and cooled for 10 min on ice, and then incubated at 26°C for 30 min. Next, 200 µL of a 1/1000 dilution of streptavidin-POD (GE Healthcare) were added to the individual wells and developed using ABTS solution as above.

To study the sensitivity of the aptamers to recognize LiH2A from *L. infantum* promastigotes, lysates (cytoplasmic plus nuclear fractions) corresponding to increasing number of parasites (1-10^5^) were plated in coating solution (KPL) and the ELONA was developed as above with biotin-labeled aptamers at 10 nM.

### Slot blot analysis with aptamers

Two amounts of rLiH2A protein (50-500 ng) and 500 ng of BSA were transferred onto nitrocellulose membranes under vacuum. Filter were washed three times in PBS-T (10 mM sodium phosphate, 0.15 M NaCl, 0.05% Tween-20, pH 7.5) for 10 min and then blocked with 5% milk in PBS-T for 1 h at room temperature. Afterwards, membranes were incubated with digoxigenin-labeled aptamers at 1 µg/mL (40 nM) in selection buffer for 1 h at room temperature with gentle rocking. After the designated time, membranes were washed with selection buffer three times and probed with anti-digoxigenin-POD antibody diluted 1/1000, for 1 h at room temperature. Excess enzyme was removed by three subsequent washes with selection buffer. Finally, the membranes were developed with enhanced chemiluminescence’s kits (GE Healthcare) and exposed to hyperfilm. BSA was used as negative control.

### Immunoblotting

Thirty μg of *L. infantum* total (lane T), cytosolic (lane C) and nuclear (lane N) proteins and 1 μg of recombinant LiH2A (lane R) were separated on 15% SDS-PAGE gels and transferred to nitrocellulose membranes. Membranes were incubated with 1 µg/mL (40 nM) of the digoxigenin-labeled AptLiH2A#1 and AptLiH2A#2 in selection buffer for 1 h at room temperature with gentle rocking and then washed with selection buffer three times and probed with anti-digoxigenin-POD antibody diluted 1/1000, for 1 h at room temperature. Finally, after three subsequent washes with selection buffer, the membranes were developed as above.

### Mapping of the LiH2A-aptamer interaction

Interaction of aptamers with LiH2A was studied as previously reported [26]. Briefly, nine peptides corresponding to overlapped sequences of *L. infantum* H2A protein (10 μg/well) were incubated with digoxigenin-labeled AptLiH2A#1 and AptLiH2A#2 at 0.5 µg/mL (20 nM) in 96-well microtiter plates at 26°C for 30 min. After washing, anti-digoxigenin-POD antibody was added and developed using ABTS solution. Peptides were synthesized by the simultaneous multiple solid-phase synthetic method using a polyamine resin and FMOC chemistry [30] and purity was checked by amino acid analysis and HPLC chromatography. The analysis of the secondary structure of the peptides was performed using the PSIPRED v3.3 software (http://bioinf.cs.ucl.ac.uk/psipred/) developed by the Bioinformatics Group, headed by Professor David Jones, within the Department of Computer Science at University College London.

### Aptamer pull-down assay

To test the ability of individual aptamers to specifically bind to and purify LiH2A presents in lysates, purification experiments using streptavidin magnetic microparticles were carried out.

Streptavidin-conjugated magnetic microparticles (Sigma) were incubated for 30 min at 4°C with stirring in selection buffer and 1 mg/ml of cytochrome C (Sigma) to block nonspecific binding. Then, 30 µg (1.2 nmol) of biotin-labeled aptamers prepared as above were incubated with 1 mL of 50% streptavidin-conjugated microparticles (Sigma) for 1 h at 4°C with shaking. Next, lysates from *E. coli* cells overexpressing LiH2A (50 μg) or fraction enriched in nuclear proteins from *L. infantum* (100 µg) were added to the microparticles:aptamer complexes and incubated for 30 min at 26°C. Finally, samples were placed on the magnet until pellet forming and the supernatant was discarded. Then, pellet was washed three times with selection buffer, resuspended in 3x loading buffer, resolved by 15% SDS-PAGE and silver stained.

### Statistical analysis

Data are presented as an average value ± standard error of the mean (SEM) from three to six independent measurements in separate experiments and analyzed using GraphPad Prism v4 (San Diego, CA, USA). The statistical significance was performed by analysis of variance ANOVA followed by Dunnett’s test.

## Results

### Isolation and structural characterization of aptamers against LiH2A

In previous studies in our laboratory, we selected and characterized a DNA aptamer population that recognized *L. infantum* H2A histone (LiH2A) with very high affinity and specificity [26]. In the present paper, we have isolated and identified aptamer sequences that strongly bind LiH2A from the SELH2A population previously obtained after 3 rounds of selection (Rd3) to determinate whether or not these aptamers could be used as biorecognition molecules and laboratory tools.

**Figure 1:**
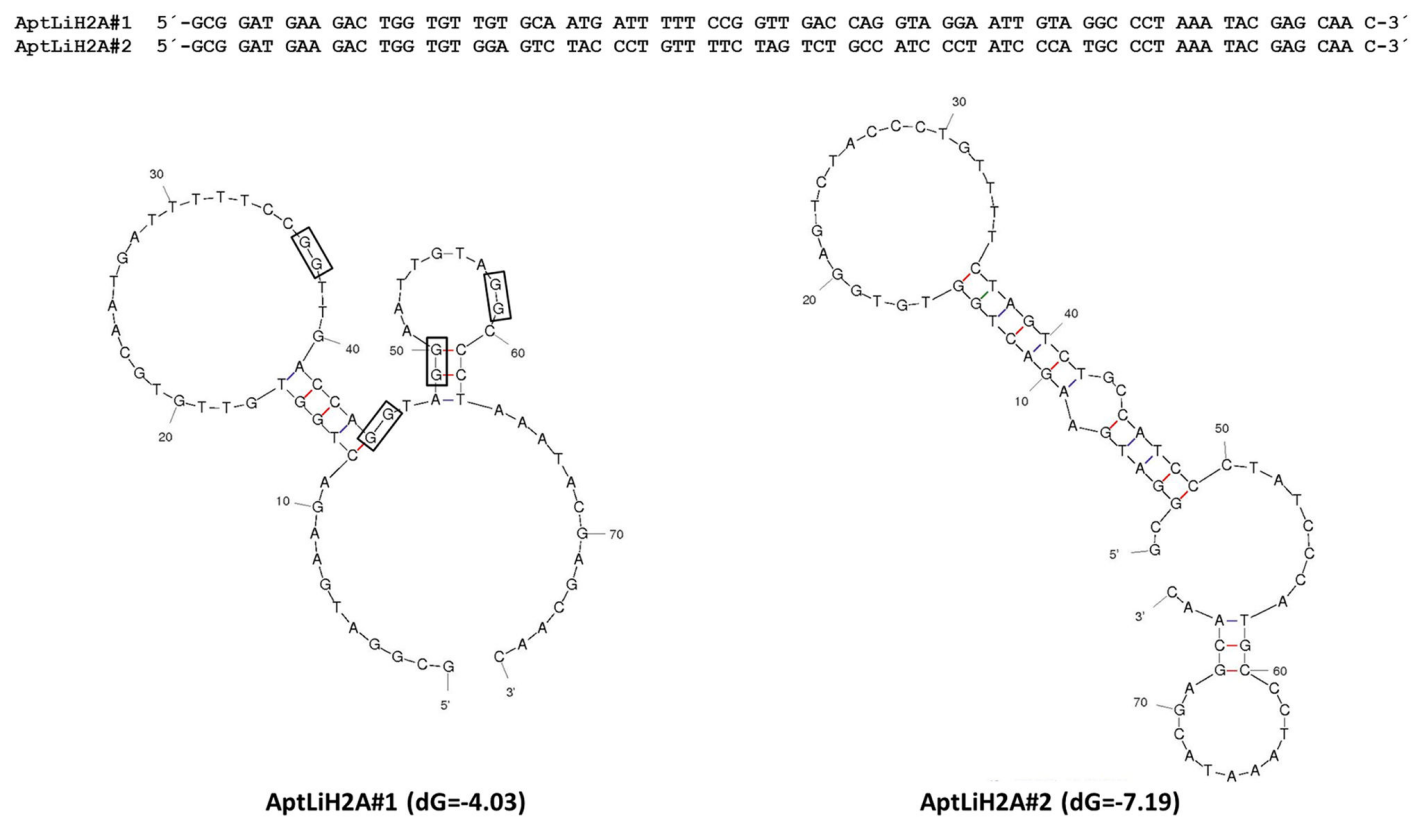
Sequence and secondary structures of selected aptamers. The DNA sequences of AptLiH2A#1 and AptLiH2A#2 were analyzed using ‘mFold’ software. The resultant secondary structures with the lowest free energy folding are shown. The boxed G-doublets are those with the highest probability (G-Score) of participating to the G-quadruplex formation, based on the algorithm used by the QGRS Mapper software.

With this purpose, aptamer population was cloned in a T-vector plasmid and 30 clones were isolated and sequenced. In a preliminary study, the affinities of all these individual anti-LiH2A aptamers for their target were estimated by ELONA and the results indicated that two of these clones, named AptLiH2A#1 and AptLiH2A#2, were able to detect LiH2A with the highest affinities relative to the control (data not shown). These two aptamers were chosen for further characterization of their structures and ability to bind specifically the target from lysates. Aptamer sequences were obtained and then analyzed using the program mFold, which yielded one potential secondary structure for each of the aptamers in the same condition that those used during selection (Figure 1). As it can be observed, both sequences show complex secondary structures, including protruding loops and stems. Taking into consideration the Gibbs free energy (dG) value, AptLiH2A#2 (dG=-7.19) may adopt a more stable structure than AptLiH2A#1 (dG=-4.03). Furthermore, a QGRS-mapper was used to predict putative G-quadruplexes formed from G-rich sequences in aptamers, revealing a high possibility of the presence of a G-quadruplex structure in AptLiH2A#1, which may confer a higher stability to this structure.

Typical stem and loop motifs, similar in both aptamers, although located in different positions in each of them, can be seen in their secondary structure (Figure 1). However, AptLiH2A#1 shows a higher percentage of guanine nucleotides (30.2%) than AptLiH2A#2 (21.0%), which are spaced as G doublets with potential ability to form G-quadruplexes. 

### Affinity characterization of aptamers against LiH2A

**Figure 2:**
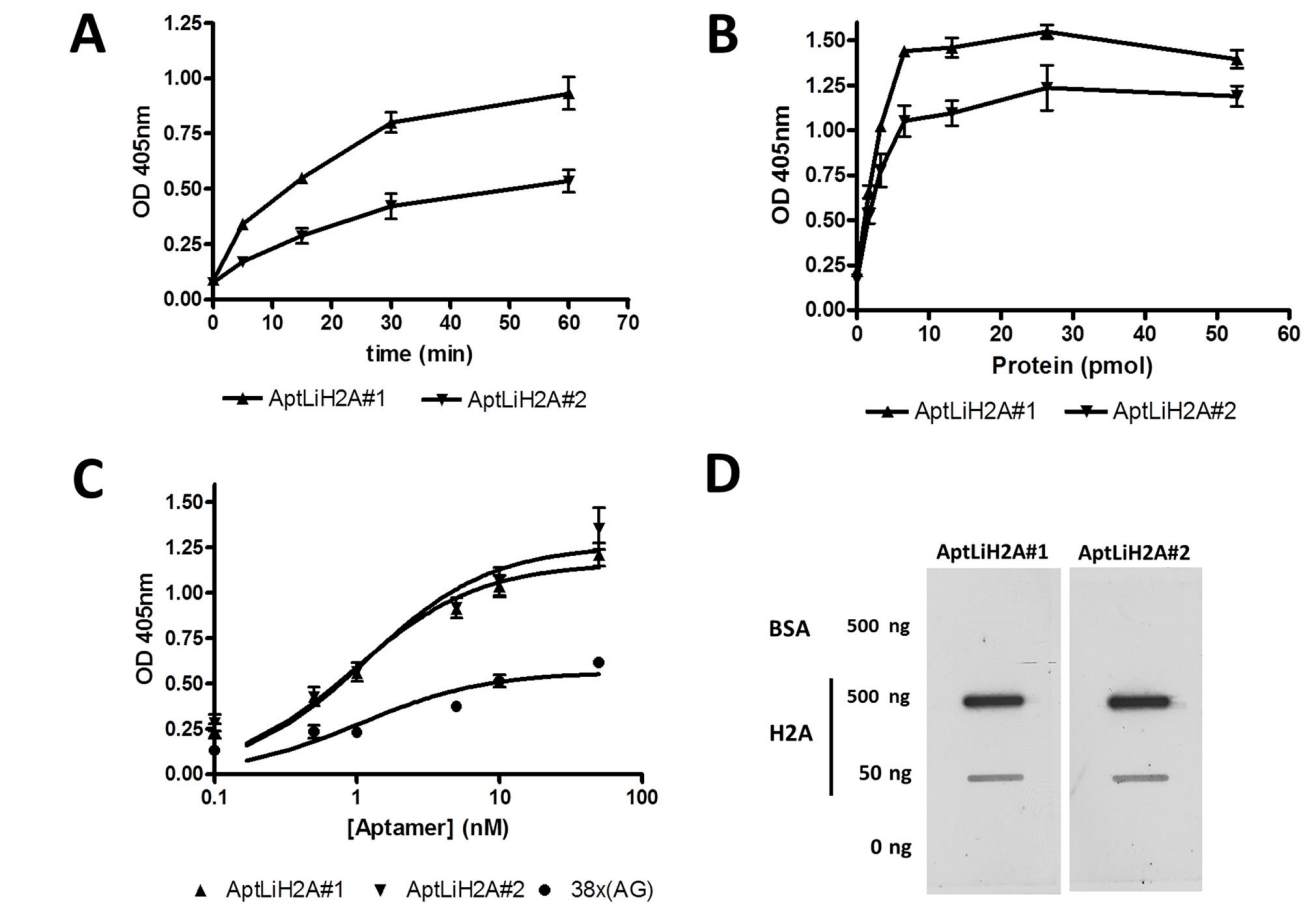
Binding affinity of AptLiH2A#1 and AptLiH2A#2 for *L. infantum* H2A protein. (**A**) Binding kinetics of AptLiH2A#1 and AptLiH2A#2 to LiH2A by ELONA. Recombinant LiH2A protein was plated, incubated with digoxigenin-labeled aptamers at the concentration indicated in “Materials and methods” for 5, 15, 30 and 60 min. Finally, anti-digoxigenin-POD antibodies were added and revealed with ABTS solution at 405 nm. (**B**) Several amounts of rLiH2A (0-52.8 pmol) were incubated with digoxigenin-labeled AptLiH2A#1 and AptLiH2A#2 and revealed as above. (**C**) Recombinant LiH2A protein was plated at 13.2 pmol/well and incubated with several concentrations (0–50nM) of biotin-labeled AptLiH2A#1, AptLiH2A#2 and 47x(AG) aptamers. Afterwards, streptavidin-POD was added and revealed as above. All the experiments were made in triplicate and average of two different experiments is shown. All the ELONA experiments were made in triplicate and average of 4-5 different experiments is shown. (**D**) BSA and rLiH2A at the concentration indicated in the figure were fixed under vacuum to a nitrocellulose membrane. Membranes were blotted with digoxigenin-labeled AptLiH2A#1 and AptLiH2A#2. Afterwards, the membrane was probed with anti-digoxigenin-POD antibodies, developed with enhanced chemiluminescence’s kits and exposed to hyperfilm. The membrane shown is representative of at least three different experiments.

In order to determinate the time necessary to reach the highest binding of aptamers to LiH2A, we have determined the binding kinetics of AptLiH2A#1 and AptLiH2A#2 in an ELONA assay as described in Materials and Methods. Thus, 66 pmol of recombinant LiH2A (rLiH2A) were incubated with digoxigenin-labeled AptLiH2A#1 or AptLiH2A#2 (20 nM) for 5, 15, 30 and 60 min and absorbance at 405 nm was determined after incubation with anti-digoxigenin-POD antibody. Results clearly indicated that AptLiH2A#1 and AptLiH2A#2 bound to rLiH2A protein in a time-dependent manner (r=1; P<0.0001; Spearman correlation) reaching a plateau after 60 min of incubation (Figure 2A). In view of these results, we consider that 30 min incubation is time enough to get most of the aptamers bound to the target. 

Next, to determine the lowest amount of protein necessary to reach the highest binding, we performed an analysis in which 96-well plates were coated with quantities ranging from 25 ng to 800 ng (1.65 pmol to 52.8 pmol) and incubated in the presence of 5 nM digoxigenin-labeled AptLiH2A#1 or AptLiH2A#2 as described in Materials and methods. The results showed that both aptamers detected rLiH2A protein in a concentration-dependent manner and that the affinity to the aptamer was directly proportional to the quantity of rLiH2A until 100 ng of protein for both AptLiH2A#1 and AptLiH2A#2 aptamers (Figure 2B).

In order to study the binding capacity of aptamers AptLiH2A#1 and AptLiH2A#2 for H2A protein, we performed an ELONA assay in which the 96-well plates were coated with rLiH2A protein (200 ng/well) and several concentrations of biotin-labeled AptLiH2A#1, AptLiH2A#2 and a non-specific linear 38x(AG) aptamer were tested as described in Materials and methods. Data were analyzed using non-linear regression showing that they responds to a one-site binding curve with an equation y = (x × Bmax)/(x + K_D_) where Bmax is the maximal binding and K_D_ is the concentration of ligand required to reach half-maximal binding. As it can be observed in Figure 2C, the specific aptamers against LiH2A reach a much higher signal (Bmax = 1.16 ± 0.05 U and 1.26 ± 0.07 U) than the control 38x(AG) aptamer (Bmax = 0.56 ± 0.04 U). From the same results, we concluded that AptLiH2A#1 and AptLiH2A#2 aptamers are able to detect rLiH2A in a concentration-dependent manner with K_D_ = 0.96 ± 0.17 nM and 1.16 ± 0.28 nM, respectively. 

Finally, we decided to study the binding of selected aptamers to the target using an alternative technique. Therefore, we performed slot blot experiments in which two amounts of rLiH2A protein and BSA were transferred onto nitrocellulose membranes and the immobilized proteins were probed with a 40 nM concentration of both digoxigenin-labeled AptLiH2A#1 or AptLiH2A#2 as described in Materials and methods. As shown in Figure 2D, both aptamers showed strong binding to rLiH2A protein but did not show any binding to negative control BSA, thereby indicating their specificity towards rLiH2A target. 

All the above results clearly indicate that both aptamers, AptLiH2A#1 and AptLiH2A#2, are able to bind the rLiH2A protein efficiently, supporting the possibility of use these aptamers as recognition molecules in a diagnostic system.

### Specificity of the aptamers against LiH2A

**Figure 3:**
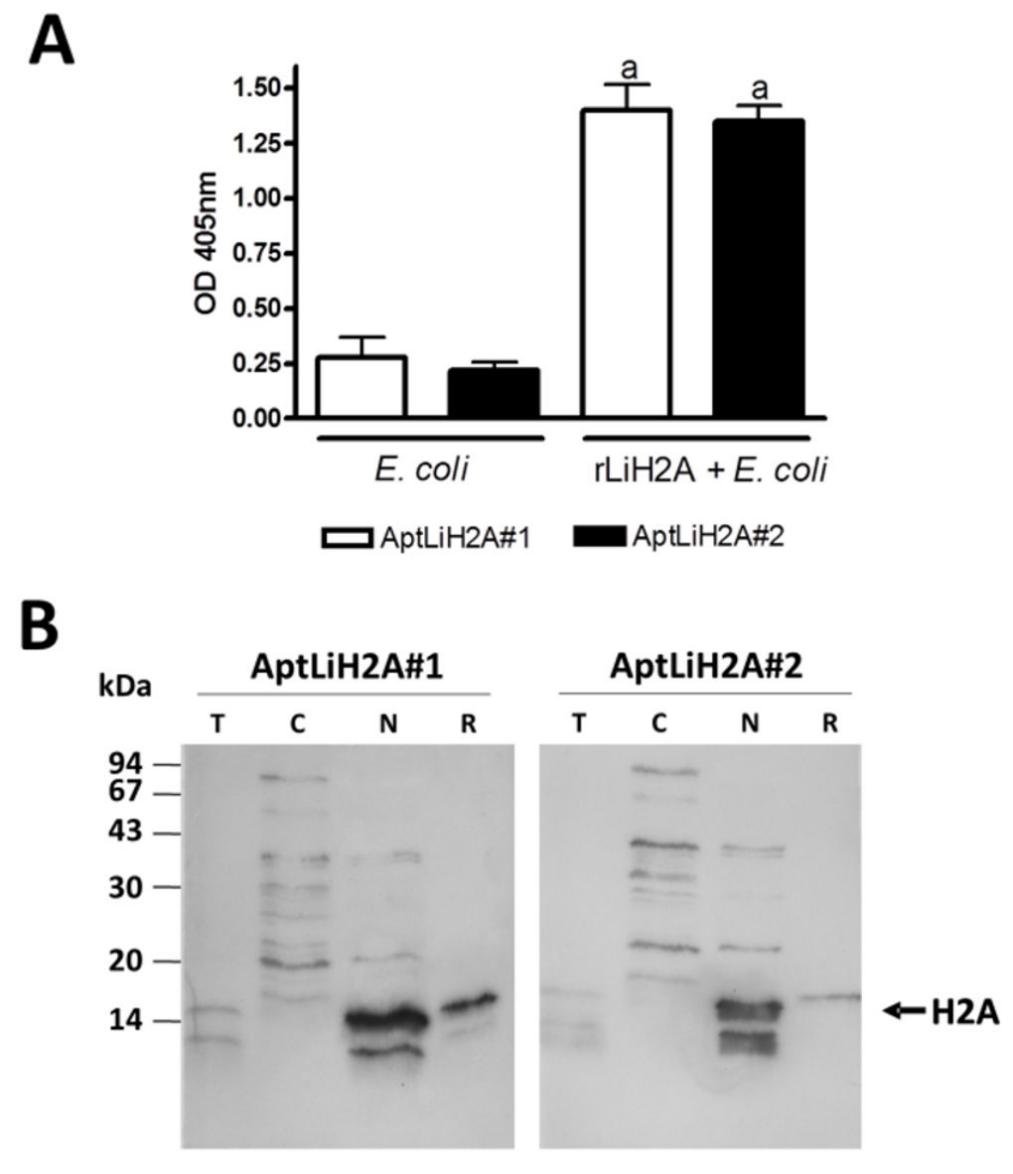
Specificity of the aptamers against LiH2A. (A) Lysates from *E. coli* bacteria expressing rLiH2A (*E. coli* + LiH2A) or from untransformed *E. coli* bacteria were plated at 1 µg/well and incubated with 10 nM of biotin-labeled AptLiH2A#1 and AptLiH2A#2 and ELONA assays were performed as in Figure 2C. All the experiments were made in triplicate and average of three different experiments is shown. Statistical significance was calculated between *E. coli* and the value obtained for *E. coli* expressing rLiH2A (a, p<0.001). (B) *L. infantum* total (lane T), cytosolic (lane C) or nuclear (lane N) proteins and recombinant H2A (lane R) were subjected to western blot and incubated with digoxigenin-labeled AptLiH2A#1 and AptLiH2A#2 as indicated in Materials and Methods. The membrane shown is representative of at least three different experiments.

In order to study the specificity of the selected aptamers to rLiH2A, we have first performed ELONA assays using as target lysates from *E. coli* bacteria expressing rLiH2A (*E. coli* + LiH2A) or from untransformed *E. coli* bacteria. As shown in Figure 3A both aptamers recognize rLiH2A induced in bacteria with high affinity while the values obtained when the same protein amount is plated from untransformed *E. coli* were very low (~ 5-fold lower) indicating that the aptamers are very specific for rLiH2A relative to proteins from bacteria. 

In addition, to determine the specificity of the aptamers on the endogenous LiH2A, we performed Western blot assays in which total protein and cytoplasmic and nuclear fraction from promastigotes of *L. infantum* and rLIH2A protein as a positive control were immobilized on PVDF membranes, and then probed with digoxigenin-labeled AptLiH2A#1 or AptLiH2A#2 aptamers and detected with anti-digoxigenin-POD antibody. As shown in Figure 3B, the selected-aptamers specifically recognized endogenous LiH2A in lanes corresponding to both total proteins and nuclear fraction from *L. infantum* promastigotes but, however, this band did not appear in cytoplasmic fraction. The intensity of the rLIH2A protein band can be used as a reference.

### Identification of the regions on LiH2A recognized by aptamers

**Figure 4:**
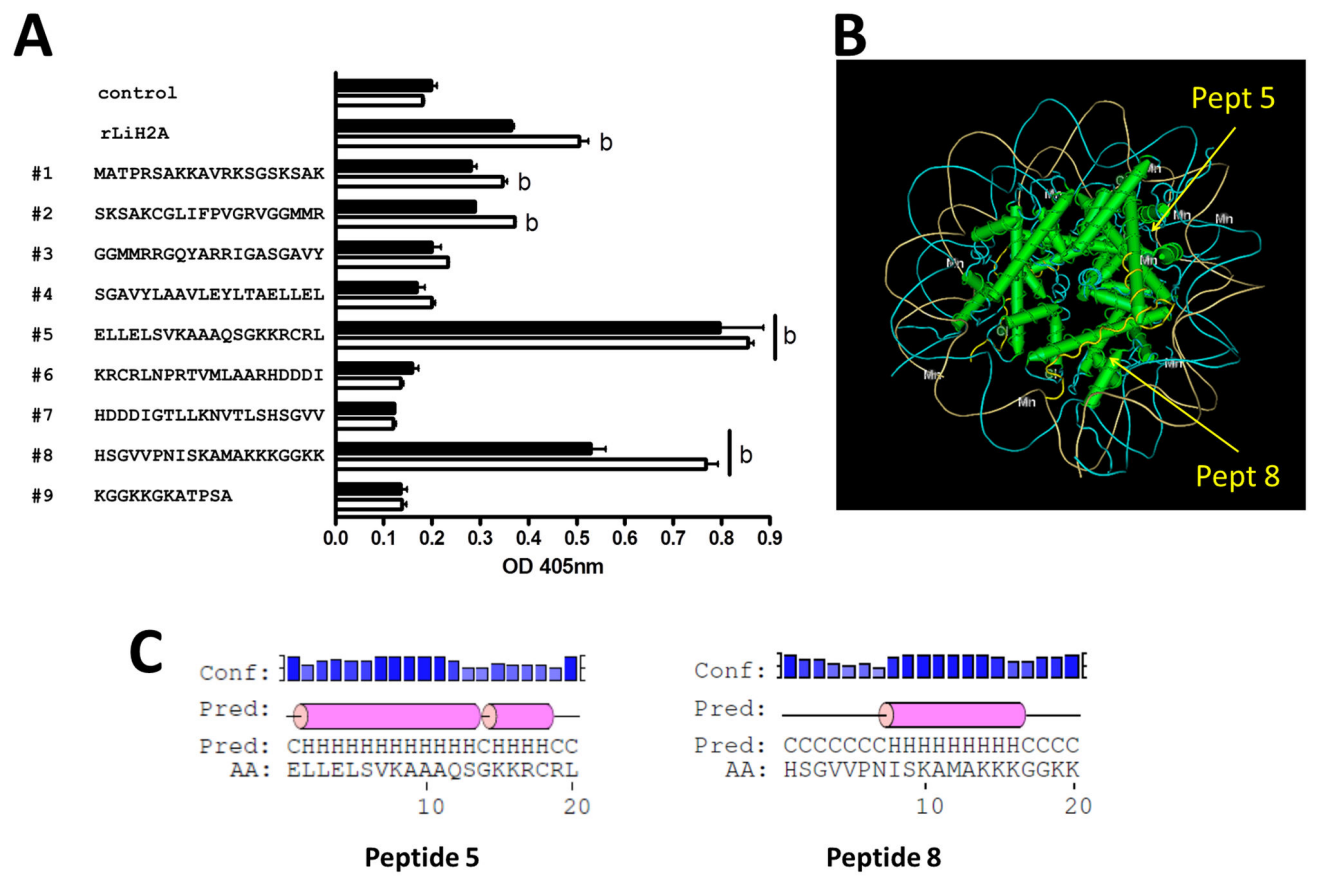
Mapping of the LiH2A-aptamer interaction. (**A**) Sequence of the peptides corresponding to overlapped sequences of LiH2A protein and ELONA assay showing the binding ability of aptamers to the different peptides. All the experiments were made in triplicate and average of four different experiments is shown in the figure (b, p<0.01 relative to the control). (**B**) Localization of the peptides recognized by AptLiH2A#1 and AptLiH2A#2 on the tertiary structure of the nucleosome. Modeling was made using Cn3D macromolecular structure viewer. (**C**) Predicted secondary structure of peptides 5 and 8 in solution. Secondary structure was predicted using the PSIPRED Protein Sequence Analysis Workbench (http://bioinf.cs.ucl.ac.uk/psipred/).

The determination of the LiH2A regions that are mainly recognized by AptLiH2A#1 and AptLiH2A#2 was performed by ELONA assays using nine peptides corresponding to overlapped sequences of LiH2A protein as targets (Figure 4A). Only peptides 5 and 8 were recognized by AptLiH2A#1 and AptLiH2A#2 with very high affinity (P<0.01; ANOVA followed by Dunnett’s test). Additionally, peptides 1 and 2 were also recognized with lower affinity although with the same significance (P<0.01) by AptLiH2A#1. Next, we decided to locate the peptides on the tertiary structure of the nucleosome. Figure 4B shows the position occupied by peptides 5 and 8, which are positioned forming a pocket region located at a side of the protein that would be more accessible to the aptamers.

### Sensitivity of the aptamers to recognize LiH2A from *L. infantum* promastigotes

**Figure 5:**
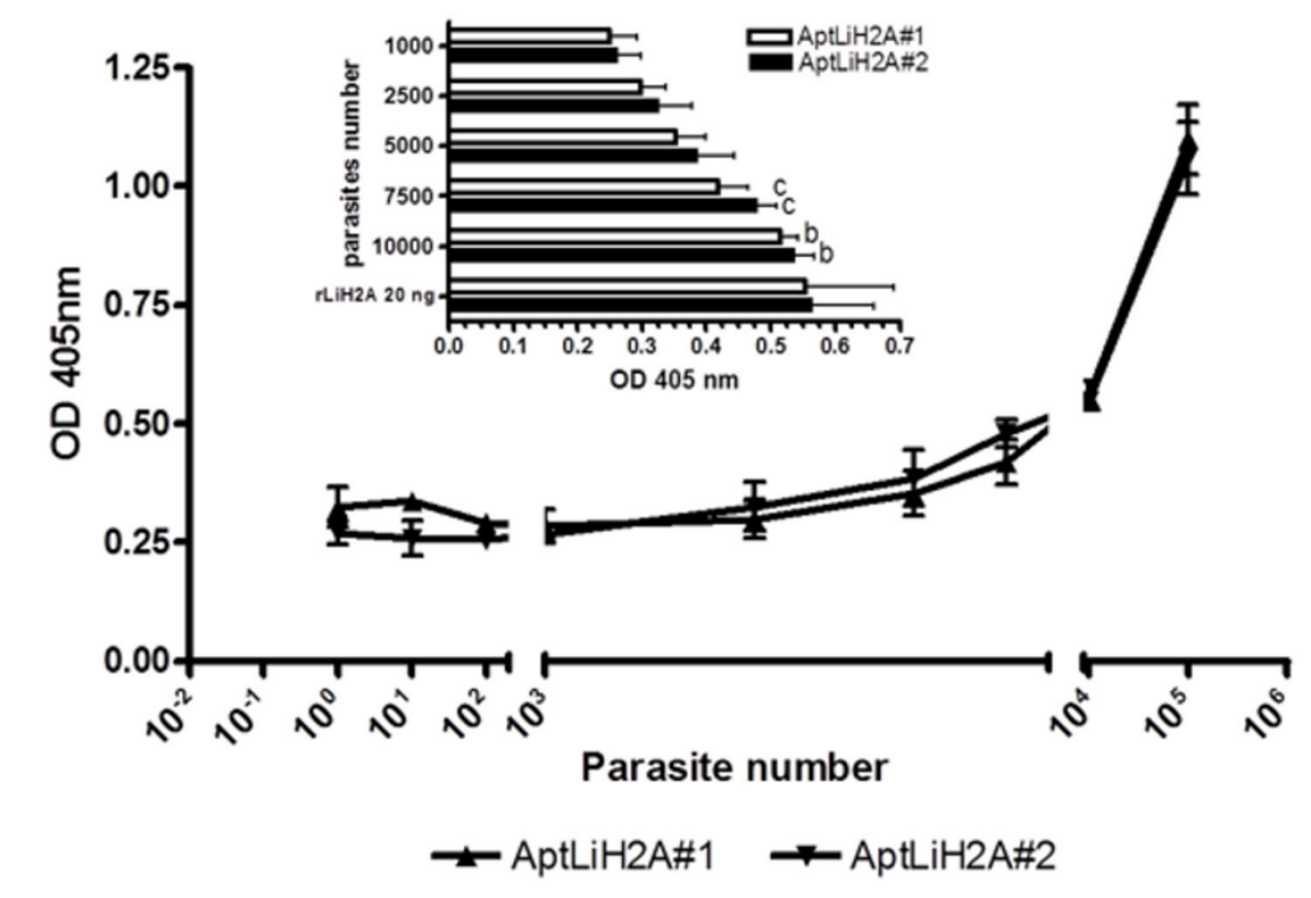
Sensitivity of the aptamers to recognize LiH2A from *L. infantum* promastigotes. Total lysates corresponding to increasing amounts of parasites (1-10^5^) were plated and incubated in the presence of biotin-labeled AptLiH2A#1 and AptLiH2A#2 at 10 nM and ELONA assays were performed as in Figure 2C. All the experiments were made in triplicate and average of 3-5 different experiments is shown. Statistical significance was calculated between each parasite number and the value obtained for 1000 parasites (b, p<0.01; c, p<0.05).

In order to determinate the lowest number of *L. infantum* promastigotes present in a sample detected by AptLiH2A aptamers, we performed ELONA assays in which total lysates corresponding to increasing amounts of parasites (1-10^5^) were plated and incubated in the presence of AptLiH2A#1 and AptLiH2A#2. The results showed that both aptamers were able to detect LiH2A from 10000 promastigotes whereas that the LiH2A protein from 1000 parasites was almost undetectable (Figure 5). In view of this result, it was decided to perform a further test using various amounts of promastigotes in the range 10^3^-10^4^ in order to quantify more securely the number of detectable parasites (Figure 5, inset). The results indicated that both aptamers are able to detect LiH2A protein from 7500 parasites in a significant way.

### Protein isolation from cell lysates

**Figure 6:**
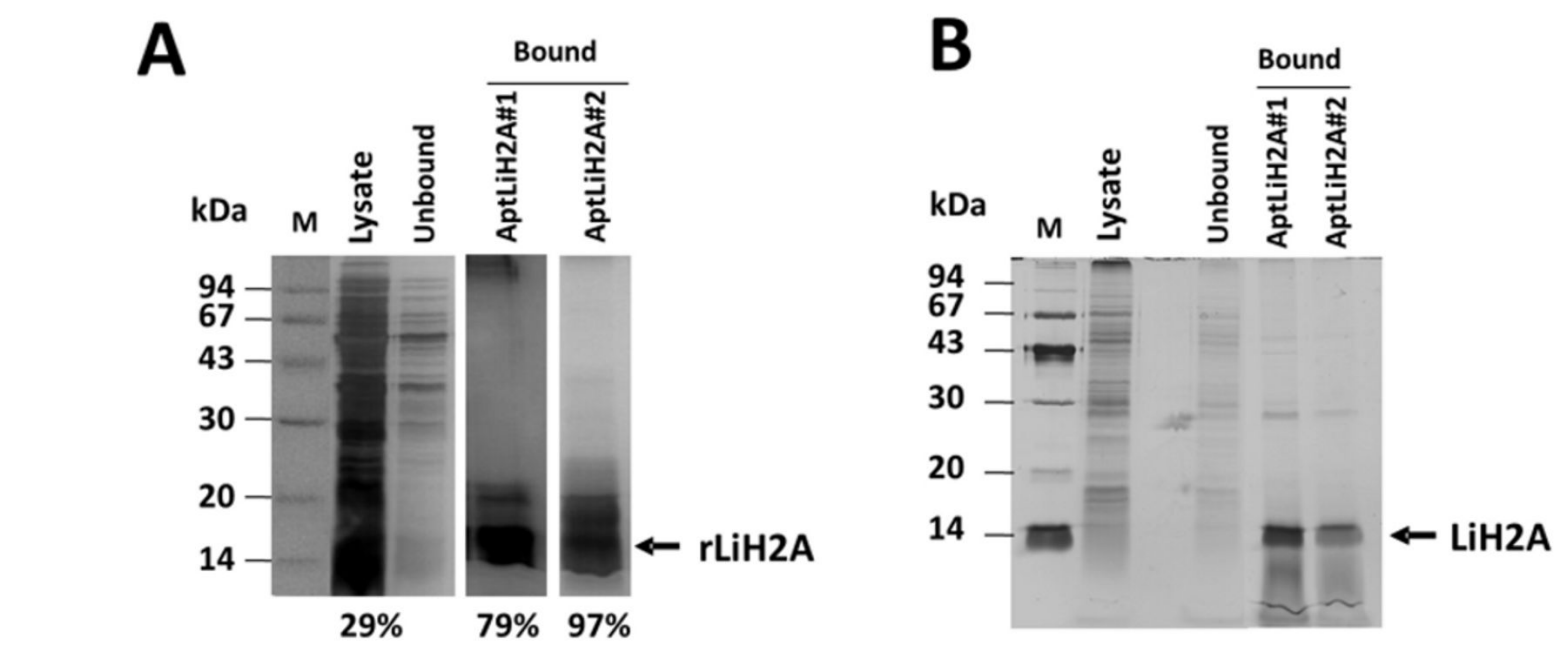
Aptapurification of LiH2A. Biotin-labeled AptLiH2A#1 or AptLiH2A#2 bound to streptavidin-coated magnetic beads were incubated with lysates from HIS-LiH2A-expressing *E. coli* bacteria (**A**) or from *L. infantum* promastigotes (**B**). The unbound fraction of the lysates was removed and the target protein analyzed by 15% SDS-PAGE and visualized by silver stain. A band corresponding to LiH2A in both aptamers-bound fractions is observed. The analysis of the data shown an enrichment in HIS-LiH2A from 29% to 79% and 97% after using AptLiH2A#1 or AptLiH2A#2, respectively (**A**) and that both aptamers are able to increase the relative amount of endogenous LiH2A after purification about 90% (**B**). Silver stained gels are representative of three different experiments.

One of the most important applications of the aptamers is as laboratory tools detecting or purifying proteins for its further characterization. In order to study whether or not some of the selected aptamers is/are capable to bind to recombinant LiH2A, biotin-labeled AptLiH2A#1 or AptLiH2A#2 were bound to streptavidin-coated magnetic beads for 1 h. Next, lysates from HIS-LiH2A-expressing *E. coli* bacteria were incubated with microparticles:aptamer complexes for 30 min at 26°C. The unbound fraction of the lysates was removed and the target protein analyzed by 15% SDS-PAGE and visualized by silver stain. As shown in Figure 6A, a band corresponding to LiH2A in both aptamers AptLiH2A#1- and AptLiH2A#2-bound fractions were observed indicating the high affinity of these aptamers for LiH2A. The analysis of the data showed an enrichment in HIS-LiH2A from 29% in the *E. coli* lysate after IPTG treatment to 79% and 97% after purification with aptamers AptLiH2A#1 or AptLiH2A#2, respectively.

In other set of experiments, we analyzed the possibility to employ AptLiH2A#1 or AptLiH2A#2 to purify endogenous LiH2A from lysates from *L. infantum* promastigotes (Figure 6B). The results demonstrated that both aptamers were able to increase very significantly the relative amount of LiH2A (around 90%). All together, these results clearly demonstrate that both aptamers can be reliably used for purification of LiH2A from parasites lysates.

## Discussion

Trypanosomatide parasites produce a great deal of chronic diseases affecting hundreds of millions people, mainly in underdeveloped countries. Among these diseases, *Leishmania* produces a devastating human disease, leishmaniasis, which severely affects around 12 millions of people in Asia, Africa and South America [2]. The development of new diagnostic and therapeutic tools against leismaniasis is imperative, and consequently, mobilization of financial resources to fund research on diagnosis and randomized controlled trials of treatment should be international health priorities.

During the last years, in our laboratory we have selected aptamer populations that specifically recognized *Leishmania* proteins [25-27]. Our aim is to include these aptamers in developing detection/therapeutic systems for leishmaniasis and also as laboratory tools. Accordingly, in a previous study [26], *L. infantum* H2A antigen was selected as a target because this protein is considered an excellent candidate for leishmaniasis diagnosis [31] and therapy [32]. In that study, we selected an aptamer population, named SELH2A, which showed high specificity for LiH2A but no cross reactivity to BSA or others *Leishmania* proteins. In fact, we demonstrated that SELH2A was able to identify native H2A in a total lysate containing all the parasite proteins with a very low background. These results strongly supported the use of aptamers in currently employed diagnostic systems (ELONA or Western blot).

In this paper, we isolate and characterize individual aptamers from the previously obtained SELH2A aptamer population in order to check the possibility of using those aptamers for detection of LiH2A and, more important from a researching point of view, like laboratory tools. As a result, we described two individual aptamers which bind to LiH2A with affinity and specificity comparable to those reported for the previously selected population [26]. In fact, both AptLiH2A#1 and AptLiH2A#2 recognize rLiH2A protein with an affinity constant in the very low nM range. These values are in the best range of most of the aptamers previously described [33].

 Histones are highly alkaline proteins that, through the positive charges of the side chains of the basic aminoacids, bind to DNA phosphate groups (negatively charged), for it is not relevant the base sequence of DNA. In this regard, it should be noted that the aptamers are nucleic acids and therefore may interact non-specifically with histones. Therefore, an aptamer whose sequence consists of 38 pairs (AG), which has the same charge as the aptamers against LiH2A but that is not capable of forming any secondary or tertiary structure, is included in the assays as a control. The results clearly show that the selected aptamers give a much higher signal than the control aptamer, indicating that the recognition not only depends on the charge of the nucleic acid but also of three-dimensional structure that aptamers are capable to adopt, allowing specific interaction with concrete regions of the histone.

Interestingly, the sequence and the secondary structure of these aptamers are very different although both aptamers recognize the same peptides in the LiH2A sequence (peptides 5 and 8). These results clearly suggest that each of these aptamers might adopt a different tertiary structure but that both are able to interact with the same region of the target. Our data also suggest that AptLiH2A#1 and AptLiH2A#2 are very stable molecules. However, dG values of their more probable secondary structures are different. The analysis of the secondary structures obtained using mFold software shows that the conserved regions of AptLiH2A#2 probably contribute to stabilize the secondary structure of the aptamer, which is reflected in a lower dG. On the other hand, AptLiH2A#1 shows a higher dG, calculated taking into account the hydrogen bonds between base pairs but, however, it may have a high stability due to the presence of a G-quadruplex structure. 

As other parasites, diagnosis of diseases caused by Trypanosomatids is currently dominated by microscopic examination or serodiagnosis meanwhile others diagnostic techniques, such as PCR or LAMP, are still expensive and not widely accessible in many countries. In addition, soon after a natural infection, or during the chronic phase of the trypanosomatid diseases, the number of parasites in blood is usually very low and, in consequence, difficult to detect by PCR or LAMP. During the last decade, many efforts have been addressed to develop diagnostic tools using aptamers as recognizing molecule. At this respect, several previously published papers strongly support that although the antibody is the gold standard for protein detection and identification, aptamers can compete with antibodies as biorecognition elements in different clinical and biotechnological detection system [34,35] due to easier and lower cost of their production and the feasibility of the aptamer-based diagnostic system. In this sense, aptamers against surface molecules from *Leishmania* may enable direct detection of the parasite instead of their cognate antibodies.

In 2007, Bruno et al [36] reported the development of several DNA aptamers against surface molecules from *Leishmania donovani* promastigotes, which showed quantitative binding data in ELONA assays. Several of these aptamers exhibited very high affinity against *L. donovani* and, at lesser extent, *L. tropica*. Very interestingly, these authors demonstrated that the aptamers showed evidence of “hook effect” at high target concentration. Most recently, Operational Technologies Corporation (OpTech) has completed a successful Phase I SBIR contract in which 72 new candidates DNA aptamer sequences against *Leishmania major* were developed and screened by ELISA-like plate assay (http://www.sbir.gov/sbirsearch/detail/383773).

Following the same approach, our results confirm that aptamers targeting LiH2A are capable to detect the amount of histone corresponding to around 7500 parasites using ELONA platform. Obviously, this detection limit is far from that desired for a diagnostic system. However, in our laboratory, we are working on the development of a more sensible system also based on ELONA, which may be potentially used as a novel diagnostic tool.

One of the main aims of this work was to study the possibility of using the aptamers to purify LiH2A from complex mixtures as has been previously reported for other targets [37]. The feasibility of the method was demonstrated on a recombinant form of LiH2A overexpressed in *E. coli* bacteria and using lysates from *L. infantum* promastigotes, but we anticipate that it could be used for any other protein for which aptamer development is possible. Our results clearly demonstrate that both aptamers are capable to interact and purify rLiH2A and also the endogenous LiH2A protein from *Leishmania* lysates. We consider that these aptamers may be an important tool for further characterization of the protein.

In conclusion, we have identified two ssDNA aptamers that bind with high specificity to *L. infantum* H2A protein showing very low cross-reactivity against other proteins of the parasite. Our results demonstrate that these aptamers can be used for detection of LiH2A in ELONA, slot blot and Western blot formats. Furthermore, in this paper we also demonstrate that the selected aptamers can then be used in the affinity purification of the target protein from the cell lysates. Considering the extreme complexity of cell lysate, aptapurification can be extended to potentially any biological mixture such as blood, urine and others. Aptapurification facilitates the faster generation of new recombinant proteins in pure form for their structural and functional studies as well as for other biomedical applications.

Therefore, it is reasonable to expect that these systems generated by aptamers will pave the way for future detection systems in which antibodies have been previously used. In addition, aptamers can be used as antibody surrogates in applications such as biosensors [35], fluorescent microscopy [38], or flow cytometry [22,34].
